# The strengths based approach as a service delivery model for severe mental illness: a meta-analysis of clinical trials

**DOI:** 10.1186/s12888-014-0243-6

**Published:** 2014-08-29

**Authors:** Nashwa Ibrahim, Maria Michail, Patrick Callaghan

**Affiliations:** University of Nottingham, Room B33, School of Health Sciences, Faculty of Medicine and Health Sciences, (South Block), Queens Medical Centre, Derby Road, Nottingham, NG7 2UH UK; Faculty of Nursing, Psychiatric and Mental Health Nursing Department, Mansoura University, Mansoura, Daqahlia 35516, Egypt; University of Nottingham, Room D17 Institute of Mental Health, Innovation Park, Triumph Road, Nottingham, NG7 2TU UK; University of Nottingham, Room 1977 A floor, Queens Medical Centre, Nottingham, NG7 2UH UK

**Keywords:** Service delivery models, Medical model, Strengths approach, Systematic review, Meta-analysis

## Abstract

**Background:**

The strengths-based approach is considered a paradigm shift from the deficits- focused service delivery models. The aim of this review was to systematically review randomised controlled trials (RCTs) and quasi experimental studies examining the impact of the strengths-based approach on level of functioning and quality of life as primary outcomes and psychotic symptoms as secondary outcomes in people diagnosed with severe mental illness.

**Methods:**

This review was conducted in the School of Health Sciences, University of Nottingham, United Kingdom. Participants in the primary studies were adults diagnosed with psychotic disorders. The methodological quality of the included studies was assessed independently by two reviewers using “Consort 2010” checklist for the randomized controlled trials or “TREND” for the quasi-experimental studies. The EPOC checklist was used for data extraction while management of data and meta-analysis were performed using Review Manager software (RevMan version 5.2).

**Results:**

No significant difference was found between the strengths-based approach and other service delivery models in level of functioning and quality of life. However, a significant effect on symptoms favouring other service delivery models was reported.

**Conclusion:**

Based upon evidence of moderate quality, this review suggests there is no effect of the strengths-based model of service delivery in level of functioning and quality of life in adults diagnosed with severe mental illness. The number of trials is low; therefore further evidence is required to ascertain the impact of the strengths-based approach in community mental health care.

**Electronic supplementary material:**

The online version of this article (doi:10.1186/s12888-014-0243-6) contains supplementary material, which is available to authorized users.

## Background

The strengths-based approach is a person-centred approach to caring which supports commitment to human potential for development and growth [1]. The implementation of the strengths-based approach consists of both structural and practice components which are reported to be unique to this delivery model. The structural component covers certain aspects such as low case load, low supervisors to case managers’ ratio, and the use of structured weekly group supervision to ensure adherence to the principles of the model. The practice components revolve around the use of the strengths assessment and personal recovery plans developed in collaboration between service users and practitioners. Moreover, the practice components utilise naturally occurring and existing environmental and community resources (non-mental health resources). A fidelity scale of the strengths-based approach was developed to ensure adherence of practitioners to items of the fidelity scale during the implementation of the model [2].

Published empirical studies about the strengths-based approach lack clear description of the underlying intervention. However, these studies share some commonalities regarding interventions; firstly, interventions focus on service users’ strengths and services were tailored to meet individual needs. Secondly, an emphasis on naturally occurring and existing environmental resources (supported employment, supported housing, supported education, and supported recreation) was apparent in these studies. Thirdly, service users collaborate with practitioners in setting their recovery plans. Finally, nearly 70% of service users in these published studies were not seen in the case management office, which is consistent with the principle of in-vivo (community) service users’ contact in the strengths-based approach. All these components are items in the strengths-fidelity scale [3].

Using the strengths- fidelity scale to assess fidelity of the strengths-based approach, in nearly all published studies, there is lack of clear distinction between this model and other services.

Studies comparing the strengths-based approach with other service delivery models have provided promising results regarding psychosocial health and wellbeing outcomes, hospitalization, family burden, overall physical and mental health, and psychotic symptoms [4]. However, there are no available systematic reviews with meta-analysis published in the English language to provide objective evaluation of the results of the primary studies about the strengths-based approach. In the current review, a meta-analysis of primary studies was conducted to evaluate and compare the strengths-based approach with other service delivery models on service users’ level of functioning and quality of life as primary outcomes and psychotic symptoms as secondary outcomes for people living with severe mental health illness.

### Objectives

The main objective of this review was to evaluate through meta-analysis the impact of the strengths-based approach on service users’ level of functioning and quality of life, as primary outcomes and psychotic symptoms as secondary outcomes in people living with severe mental health illness.

## Methods

This review adheres to the Updated Method Guidelines for Systematic Reviews in the Cochrane Collaboration Back Review Group [5]. Moreover, the Preferred Reporting Items of systematic Reviews and Meta-analyses (PRISMA) flow diagram was used to inform the searching process and outcomes [6].

### Inclusion criteria

#### Studies’ design

Due to the paucity of empirical studies on the strengths-based approach, a prior decision was taken by the reviewers to include both randomised controlled trials (RCTs) and quasi- experimental studies that evaluated the impact of the approach on people living with severe mental illness compared with other service delivery models. The service (strengths-based) could be delivered by psychiatrists, psychologists, psychiatric nurses, or social workers. No restriction was imposed on publication dates or the geographical location of the primary studies.

The review included studies published only in English due to feasibility as well as time and resources issues.

#### Participants

The review included studies whose participants were adults aged 18 to 65 years, diagnosed with psychotic disorders according to either the Diagnostic and Statistical Manual of Mental Disorders (DSM) [7] or the International Classification of Disease (ICD) [8], and receiving care at community mental health settings. Studies that included participants with intellectual difficulties were excluded.

#### Interventions

Studies that adhered to the fidelity of the strengths-based approach were included; fidelity refers to adherence of the practice to the guidelines. Also, studies that reported following the functional elements of the strengths-based approach developed by Charles Rapp (the model developer [9]) were included.

#### Outcomes

This review included three main outcomes; service users’ level of functioning, quality of life as primary outcomes and psychotic symptoms as secondary outcomes.

### Search methods for identification of studies

#### Electronic search

An initial scoping review of 51784 records was conducted through data based searching. The search strategy involved identification of keywords (medical subjects’ headings), synonyms, relevant theses and dissertations, and sources of grey literature. The Cochrane Library, Joanna Briggs Institute (JBI) Library database and CINAHL database were searched and no previous systematic reviews with meta-analysis in the English language covering this area (published or underway) were identified. Unpublished studies were identified and those fulfilling our inclusion criteria were included in the review. The searched data bases were:

-EMBASE, MEDLINE, PSYCHINFO, CINAHL, ASSIA, PASCAL, SOCIOLOGICAL
- ABSTRCATS, SCOPUS, WEB of SCIENCE, WEB of SCIENCE, Zetoc, ETHOS.

The Additional file 1 shows MEDLINE (Ovid) search terms that have been adapted to all data base searching.

#### Searching other sources

The screening of reference lists was done for both included and excluded studies to find any study of relevance to the review inclusion criteria. Google scholar as one of the grey literature sources was also searched.

#### Correspondence

Communication was established with Charles Rapp (the developer of the model [9]), as well as Matthew Modrcin, and Rick Goscha, to gain more insight about the strengths-based approach. The librarians at School of Social Welfare, University of Kansas (where the model was developed) were contacted to assist in the identification of further articles about the strengths-based approach or guide the search process.

### Data collection and analysis

#### Selection of studies

Screening of the abstracts of potentially relevant primary studies retrieved by electronic searching was performed by the primary reviewer (NI). In case of doubt or unavailable abstracts, full articles were inspected and articles were excluded based on filtering process of title, abstract and full text review to ensure only articles fulfilling the inclusion criteria were retained.

#### Quality assessment

Two reviewers (NI and MM) independently assessed the quality of retrieved studies using CONSORT 2010 [10], which is a 25-item checklist developed for reporting RCTs. Items report the following: title and abstract, introduction, methods (trial design, participants, interventions, outcomes, sample size, randomisation, and statistical methods), results (participants’ flow, recruitment, baseline data, numbers analysed, outcomes and estimation, ancillary analysis, and harms), discussion (limitations, generalizability, and interpretation), and other information (registration, protocol, and funding). For the non RCTs, the TREND statement [11] was used for assessing their quality. The TREND checklist consists of 22 items covering the following: title and abstract, introduction, methods (participants, interventions, objectives, outcomes, and sample size), assignment method, unit of analysis, statistical methods, results (participants’ flow, recruitment, baseline data, numbers analysed, outcomes and estimation, ancillary analysis, and adverse events), and discussion (interpretation, generalizability, and overall evidence). There were no disagreements between the primary and secondary reviewers’ quality appraisals.

#### Data extraction and management

The Cochrane Effective Practice and Organization of Care Group (EPOC) [12] data extraction checklist was used by NI and MM independently to extract data from eligible studies. This checklist allows the extraction of data from both randomised and non-randomised trials. The following information was extracted: interventions, participants, setting, methods, outcome measures, number of participants, results. Standard deviations, sample size, and means were also extracted (data were continuous).

The random effect model was used for combining data in the statistical pooling due to heterogeneity among included studies [13]. Statistical pooling was conducted by using Review Manager Software (RevMan version 5.2) with 95% confidence interval. Two reasons behind using this software were the free downloading of this software, and the first author having undergone training in the use of this software.

### Dealing with missing data

Authors were contacted in case of missing data; if the missing data was not obtained from authors (due to loss of the original data or no response being received); the reviewers included those studies in a narrative synthesis (Glover [14]; Macias et al. [15]; and Stanard, [16]) in order to avoid missing out any substantive conclusions about the strengths-based approach.

In case of missing data as in Macias et al. [17], Barry et al. [18], and Chamberlain [19]; the primary and secondary reviewers made the calculations needed to obtain the summary statistic for the continuous outcomes; standard deviations were calculated by calculating the difference in mean (MD); obtaining t values by the help of P value and degree of freedom (df); calculating the standard error (SE) from t value; calculating the standard deviation from the standard error following the guidelines in the Cochrane Handbook for Systematic Reviews of Interventions [20].

### Assessment of Heterogeneity

Due to the scarcity of empirical studies about the strengths-based approach, all eligible studies were included without considering clinical heterogeneity. However, the I-squared test was employed to provide an indication of variation among studies thought to be due to chance. Depending on the results of I-squared test; heterogeneity was interpreted depending on the following parameters:

- 0 to 40%: might not be considered important,
- 30 to 60%: might represent moderate heterogeneity,
- 50 to 90%: may represent substantial heterogeneity, and
- 75 to 100%: considerable heterogeneity [21]. In this review we visually inspected the forest plot to detect heterogeneity.

### Subgroup analysis

It was difficult to run subgroup analyses in this review as no data was provided in the primary studies about sub-groups of the population.

## Results

### Results of the search

**Figure 1:**
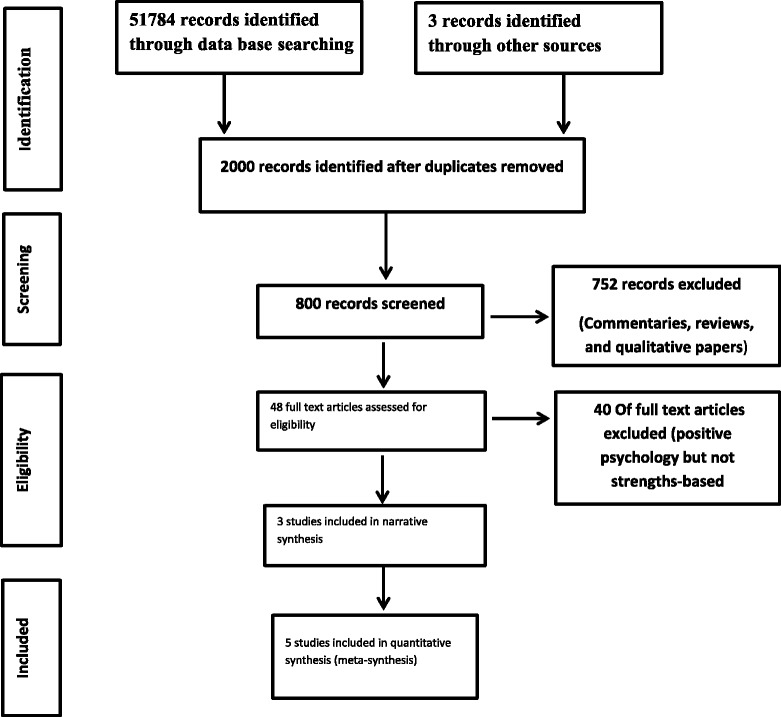
**PRISMA flow diagram of the search process.**

The PRISMA flow diagram (Figure 1) was used to report the results of the search outcomes. It shows 51784 records were screened for their titles, abstract and full text. Eight studies were identified as eligible after removing duplicates, commentaries, reviews, and qualitative studies. During the stage of quality appraisal and data extraction two quasi experimental studies and one RCT were excluded from the meta-analysis as missing data could not be obtained from the authors; two of these excluded studies (Macias et al. [15]; Stanard [16]) did not report means or standard deviations of either the intervention or the control group and upon request the authors of these papers reported that they no longer had any data. The third excluded study by Glover [14] reported no data for the control group, and we were unable to obtain the contact details for this author. Therefore, five studies were considered for pooling and inclusion in the meta-analysis; four RCTs and one quasi-experimental study.

### Description of studies included in the meta-analysis

The PRISMA flow diagram shows five studies were included in the meta-analysis (one quasi- experimental and four RCTs). A total of 194 participants from both sexes diagnosed with severe mental illness and ranging in age from 18 to 61 years were represented in these studies. It should be noted that the most common psychiatric diagnosis was schizophrenia. All studies implemented the strengths-based approach in community-based settings (in-vivo). Four of the included studies originated in the USA; one was conducted in Sweden. Additional file 2: Table S1 shows characteristics of studies included in the meta-analysis.

### Description of studies excluded from the meta-analysis

Three studies, all conducted in the USA, were excluded from the meta-analysis (two quasi-experimental and one RCT) with a total number of 232 participants from both sexes and a mean age of 43.10 years. Schizophrenia was the prominent psychiatric diagnosis among participants in those studies. Although these studies were excluded from the meta-analysis due to missing data required for calculating effect size, they did adhere to the fidelity of the strengths model. Table two shows the characteristics of the excluded studies.

### Intervention in the studies included in the meta-analysis

In Barry et al. [18] the strengths-based delivery model was compared to assertive community treatment; one professional clinician in the strength-delivery model was in charge of the service user rather than a team of health care providers as in the assertive community treatment. Moreover, the strengths-based service delivery model emphasised goal planning by the service users as well as helping them to find membership in the community through liaising with community services. Bjorkman et al. [22] provided no description of the service provided, however the study stated that the strengths-based services placed moderate emphasis on skills training, low emphasis on integration of services, and high emphasis on consumers’ engagement and input in planning the service with the case manager. The average caseload was nine clients and the service was community-based. Therefore, it can be reasonably assumed that the strengths-based components in this study adhered to the fidelity of the strengths model.

Chamberlain [19] described the functional elements of the model as: collaboration between the service user and the practitioner in the assessment of the needs of the service user in different life domains, whereby both service users’ and community strengths were assessed and determined. Mutual contracts between the service user and the practitioner were established whereby goals are client-directed and the client takes responsibility for these goals; the role of the practitioner was to help the service user to develop strategies to acquire environmental resources; social problem-solving can be achieved through resources acquisition, in-vivo tasks, cognitive restructuring, and deinstitutionalisation techniques; the strengths-based case manager is responsible for monitoring client’s achievement of the goals and changes them if needed besides providing support to the client. Macias et al. [17] found that strengths-based case managers were trained to provide linkage and brokerage of social as well as medical services. Case managers emphasised clients’ autonomy in goal planning. Strengths-based case managers were supervised; caseloads were determined to meet the fidelity of the model in terms of one-to-one contacts.

Modrcin et al. [23] implemented an intervention guided by a 250- page manual (Modrcin et al., 1985) which was not available online; a request for the manual was made to the chief author, who stated that it has been withdrawn and replaced by the *Strengths Model: A Recovery-Oriented Approach to Mental Health Services* [4], however the study provided a synopsis from this manual that might help in understanding the nature of the strengths-based intervention; “*the clients often have limited information about community services and resources; the case manager provides it. Client’s characteristically experience profound anxiety when confronting new tasks and challenges; the case manager supports and encourages. Clients frequently lack information or skills in basic aspects of independent living skills to form and sustain social relationships; the case manager offers a relationship, models and teaches social skills, and helps the client generalise that to other persons. Clients sometimes experience painful crises that place their live in turmoil and increase symptomatic behaviour; the case managers are a first line of intervention to minimise the potential for regression or relapse*” (p.308). The study by Modrcin et al. [23] reported adherence to the fidelity of the strengths model in terms of conducting weekly group supervisions and exploiting community services and resources to attain service users’ set-goals.

### Comparison (control arm) in the studies included in the meta-analysis

The strengths-based approach was compared to; assertive community treatment (ACT), the standard care services (which was described as outpatient, inpatient, and day-care facilities), and traditional community services (that were described as drug treatments and psychotherapy services), and psychosocial rehabilitation.

### Outcome measures in the studies included in the meta-analysis

The details of the outcomes which were only used in the meta-analysis are presented below:

#### Level of functioning

Barry et al. [18] used the instrumental activities of daily living (IADL) tool to assess patients’ functional level. Higher scores on this scales indicated better functioning, the joint reliability correlation for this tool ranged from 0.85-0.91. Bjorkman et al. [22] measured the psychosocial functioning using the Strauss and Carpenter Scale [24]. The Uniform Client Data Inventory (UCDI) was used by Chamberlain [19] to assess the level of functioning which consists of four subscales. As mentioned in the study, a Kappa coefficient was used to determine the reliability of this scale and the results indicated that the scale is reliable if it is used by practitioners who are familiar and have good knowledge of their clients, however, the study did not provide a measure for the reliability coefficient. In the study by Macias et al. [17] participants’ level of functioning was measured by Professional Rating of Consumer Functioning; no data was given about the reliability of this scale.

#### Quality of life

The Oregon Quality of Life Questionnaire (OQLQ) was used by Chamberlain, [19]. The OQLQ is a self-report instrument that measures service users’ perception of managing internal and external stress. The inter-rater reliability of this scale was high; above 0.80. Modrcin, et al. [23] also used the OQLQ to measure service users’ quality of life. Bjorkman et al. [22] used the Lancashire Quality of Life Profile (LQOLP) [25], but no data was given about the reliability of this scale.

#### Symptoms

In Barry et al. [18] the Brief Psychiatric Rating Scale was used to assess service users’ symptoms [26] with the reported reliability coefficient of 0.80 or greater. Bjorkman et al. [22] used the Hopkins Ckecklist-90 to assess participants’ symptoms [27] with no information provided regarding its reliability. Macias et al. [17] measured symptoms of depression, anxiety, and somatic complaints using the Brief Psychological Well-Being index. The study reported a test-retest reliability of this scale of: r (13) =0.97, p < 0.01.

### Attrition in studies included in the meta-analysis

Attrition varied considerably across studies included in the meta-analysis; in Bjorkman et al. [22] follow-up (36 months) data was available for 86% of participants. In Modrcin et al. [23] and Chamberlain [19] no data on attrition was available. In Macias et al. [17] seven participants were lost to attrition, however, five of them were replaced resulting in a final sample of 41 participants. In the quasi-experimental study by Barry et al. [18] attrition rates at two year follow-up were 88.6% for the assertive community treatment group and 67.5% for the strengths-based group. Providing a median value for attrition rate among the studies included in the meta-analysis was difficult because two of these studies did not report attrition.

### Effects of the strengths-based intervention in the studies excluded from the meta-analysis

With regard to outcomes relevant to this review, none of the excluded studies provided clear and specific information about the description of the strengths-based service delivery; Table 1, characteristics of studies excluded from the meta-analysis shows how the strengths-based interventions were described in those studies.

**Table 1:** **Characteristics of studies excluded from meta-analysis (included in descriptive synthesis)**

**Study ID/origin**	**Design**	**Intervention/comparison**	**Outcomes**	**Results**
- (Glover, 1995) [14].	-RTC.	- Three case managers received training from Charles Rapp, the	-Consumer functioning by the Utah Case Management Consumer Assessment Record.	-Study group showed significant improvement in level of functioning compared to the control group P < .01.
-USA	-Participants should havemet the definition of severe persistent mental illness to be included in the study, participants with primary diagnosis of substance misuse or organic mental disorders were excluded from the study.	developer of the strengths model as well as 20 hours of direct supervision for using the strengths model, weekly supervision meetings were held to enforce adherence to the principles of the strengths model, in addition, caseloads were 20 clients. Moreover, Case managers provided services to clients in the community. All of the former adheres to the fidelity of the strengths model scale.	-Hospital admissions were measured 42 months after implementation of the model by reviewing Valley Mental Health Records retrospectively for evidence of hospitalization 18 months before the implementation of the case management and 42 months after initiation of case management.	-At 42 months, 33% of the experimental group and 13% of the control had been hospitalised; differences between groups were not statistically significant.
	- 136 participants were randomly assigned to study and control group; 67 participants in the study group and 69 in the control.	-comparison was traditional case management services.		
(Macias, 1997) [15]	- Pre-post quasi experimental study.	-Strengths case management services were community based. Program assessment records focused on achieving clients personal goals which commit to the principles of the strengths model apparent in The fidelity of strengths modelscale.	-Quality of life and functioning variables by the Self Report inventory (Macias and Jackson, 1990) [28].	-Regarding therapists’ assessment of consumer symptomatology, strengths case management group showed significant reduction in symptoms. The MANCOVA produced significant time effect p < .01, focused *t* test showed attribution of time effect to case management on the symptoms subscale P < .001.
-USA	−97 participants were included in this study; 48 in the study group and 49 in the control group	- Comparison was treatment in usual.	-Depression, anxiety, and somatisation by the Brief Psychological Well-being (Macias and Kinney, 1990) [29].	
			-Consumer functioning by Utah Case Management Consumer Assessment Record (CCAR)	-therapists’ CCAR assessment correlates significantly with self-report measures of psychiatric symptomatology p < .05 which correlated significantly with family members’ assessment of consumer symptomatology p < .01.
- (Stanard, 1999) [16].	-Quasi experimental study.	-Case managers for the study group received 40 hours of training on the strengths model. Manipulation check was conducted to determine the effect of training on case managers, findings showed that case managers operated according to the principles of the strengths model.	- Quality of life was measured by the Quality of Life Inventory by (Frisch, 1992) [30].	-Repeated measures ANOVA on QOL showed significant interaction effect p < .05 which indicated that the experimental group showed satisfaction with QOL compared to the control group.
-USA	−44 participants took part in this study; 29 in the study group and 15 in the control group.	-strengths case management versus generalist case management	-Residential Living and Vocational Educational Status by a tool developed during strengths case management training.	-There was no significant effect regarding hospital days p > .05.
			-Hospitalisation Rate and Number of Hospital Days which was reported by case managers for 3 months before the study and during the study.	-Results showed no statistically significant differences between the study and control group regarding psychiatric symptoms p > .05.
			-Symptoms by using Hopkins Symptoms Checklist-90	-regarding hospitalisation rate before and after treatment for the whole sample, chi square test showed no statistically significant difference between both groups p > .05.
				-chi square was performed before and after treatment for residential living outcomes and it showed significance p < .001 suggesting that there was differences in residential treatment favouring the study group.

The three excluded studies varied in design; two were quasi-experimental (Macias et al. [15]; and Stanard [16]); one was an RCT (Glover, [14]). Moreover, these studies varied in the number of participants as shown in Table two. Glover ([14]) randomly assigned 136 participants to either study or control group; (67 and 69 respectively) to compare the strengths-based case management intervention with traditional case management services. Baseline measures were taken followed by six and nine months’ follow-ups; after the latter, the intervention group (the strengths-based intervention) showed significant improvement in its level of functioning, whereas the traditional case management group showed no change. Macias et al. [15] assigned 97 participants to strengths-based case management intervention as study group and community mental health centres’ services (day treatment and residential programs) as control group (n = 48 and 49 respectively). Measures were taken at baseline with nine-months follow up. Therapists’ assessment of service users’ symptomatology reported that the strengths-based management treatment significantly reduced service users’ symptomatology compared to the control group (F (1, 63) =5.42; P <0.01). Moreover, professional assessment of consumers’ symptomatology correlated significantly with both self-reports and family reports;(r = .36; p < .05); r = .47; p < 0.01).

Stanard [16] used a smaller number of participants (n = 44), with 29 receiving the strengths-based case management in the study group and 15 receiving general case management in the control group. Measures were taken at baseline for both groups and at three-months follow-up. Results of ANOVA after treatment for both study and control group showed significant effect favouring the strengths case management regarding quality of life, (F (1, 44) = 5.97, p <0.05). Regarding symptoms’ outcome, differences between both study and control group were not statistically significant.

### Risk of bias in included studies

Information about risk of bias in this review is presented in Table one.

#### Allocation

Among the RCTs included in this review, only that by Bjorkman et al. [22] described how allocation was performed by using the random number procedure in the Software Package of Statistical Analysis (SPSS); no data was provided by the other studies regarding allocation.

#### Blinding

Bjorkman et al. [22] gave no information about blinding of participants and personnel; however interviewers were blind to group allocation during outcome assessment. In the other three RCTs (Chamberlain [19]; Macias et al. [17]; and Modrcin et al. [23]), no data was mentioned about either blinding of participants and personnel or blinding of outcome assessment.

#### Reporting bias

Reporting bias is influenced by the nature and direction of results during dissemination of findings; statistically significant results are more likely to be published than non-significant results. Moreover, studies published in the English language are more likely to be published than those in other languages [20]. In this review, funnel plots of both primary and secondary outcomes were difficult to inspect visually due to the limited number of included studies. The Cochrane Handbook of Systematic reviews of Interventions [20] advises that tests be run to detect funnel plot asymmetry if the number of studies included in a review is more than 10, therefore none of these tests was conducted in this review.

#### Other sources of bias

Other potential sources of bias in this review included the variation in the control arm among the primary studies. The control delivery model used by Bjorkman et al. [22] for example was the standard care. The control group of Macias et al. [17] had undergone psychosocial rehabilitation, whereas the control arm used by Barry et al. [18] was traditional community services, described in the study as drug treatments and psychotherapy services. Additionally, the variation in sample size among included studies ranged from 41 in (Modrcin et al. [23] to 174 in Barry et al. [18]). Furthermore, the variation in the psychometric tools used to assess outcomes among included studies might contribute to bias in this review. Finally, follow-up intervals in the included studies varied between four to 36 months post-intervention (baseline). Bjorkman et al. [22] administered measures at 18 and 36 months post-intervention; Modrcin et al. [23] at four months post-intervention; and Macias et al. [17] at one year post-intervention. In the quasi-experimental study by Barry et al. [18] follow-up was administered every six months for two years, but only at four months post-intervention by Chamberlain [19].

### Effect of intervention

**Figure 2:**
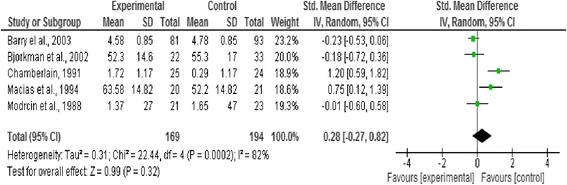
**Forest plot of level of functioning outcome.**

**Figure 3:**
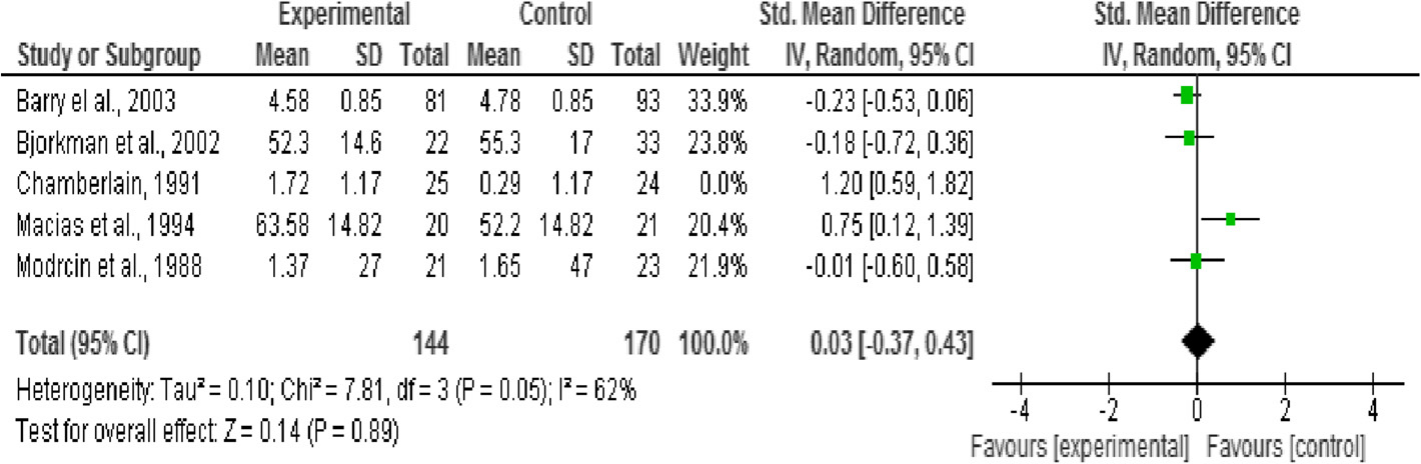
**Forest plot of level of functioning after removing (unticking or unchecking) the outlier study (Chamberlain,** **[** 19 **]** **).**

**Figure 4:**
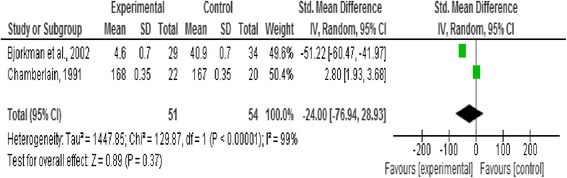
**Forest plot of quality of life outcome.**

**Figure 5:**
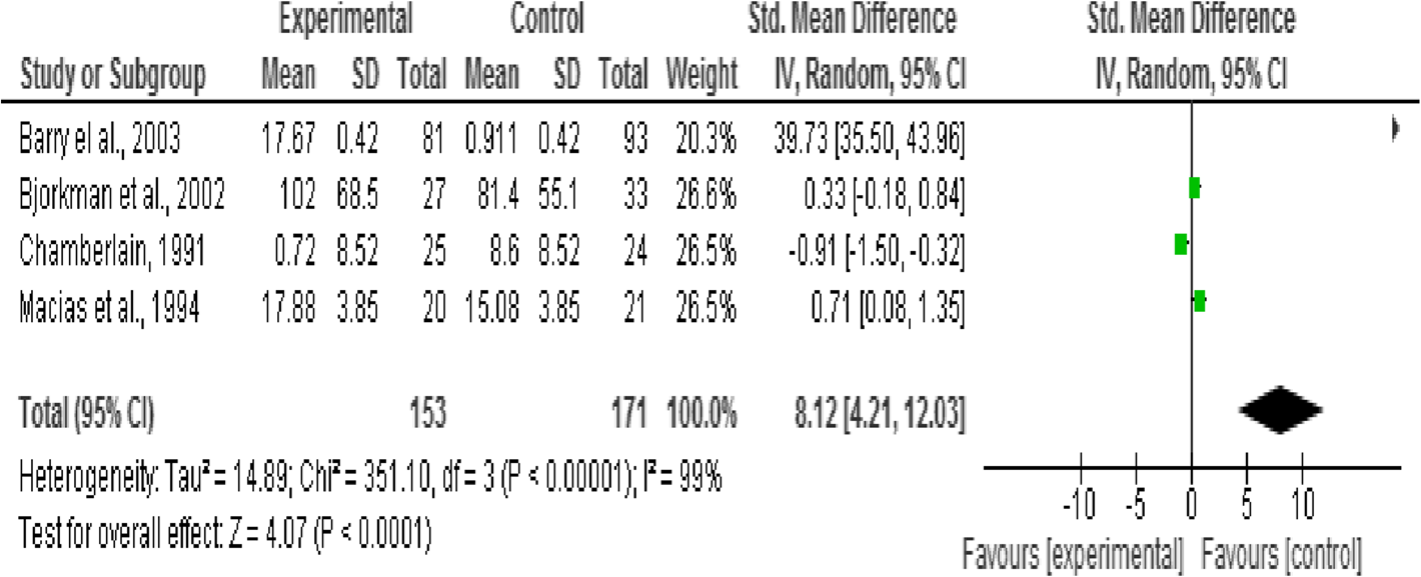
**Forest plot of symptoms outcome.**

**Figure 6:**
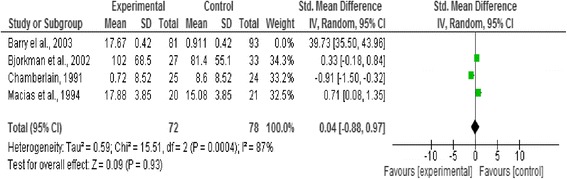
**Forest plot of symptoms outcome after removing (unticking or unchecking) the outlier study (Barry et al.** **[** 18 **]** **).**

In this review we analysed three outcome measures separately; level of functioning and quality of life as primary outcomes, and symptoms as secondary outcomes. Three forest plots presented pooled estimates for the selected outcome measures with 95% confidence intervals. Means, standard deviations, and sample size were required to calculate the effect size (Standardised mean difference) for the measurement data included, the reason behind using this effect size is the variation in the psychometric tools used in assessing the outcomes included in this review [20] (Figures 2, 3, 4, 5 and 6).

#### Level of functioning outcome

Five studies were included in measuring the overall pooled estimate for the level of functioning outcome. No significant effect was detected between the strengths-based approach and other service delivery models (n = 194 p < 0.32, CI −0.27 to 0.82) (Figure 2).

#### Quality of life

Two studies as shown in Figure four were included in the forest plot measuring the pooled estimate of quality of life. No significant effect was observed between the strengths based approach and the control group (n = 54 p < 0.37, CI −76.94 to 28.93).

#### Symptoms

This outcome was reported by Barry et al. [18], Bjorkman et al. [22], Chamberlain [19], and Macias et al. [17]. A significant effect favouring the control existed (n = 171, p < 0.0001, CI 4.22 to 12.03) (Figure five).

## Discussion

### Summary of the main results

Five studies with a total of 180 participants with severe mental illness were included in this review to evaluate the impact of the strengths-based approach as a service delivery model on service users’ level of functioning and quality of life as primary outcomes and psychotic symptoms as secondary outcomes in people living with severe mental illness. No significant difference was found between the strengths-based approach and other service delivery models regarding level of functioning and quality of life. However, a significant effect favouring other service delivery models (i.e. controls in the studies) regarding symptoms was reported, as shown in Figure five.

The substantial heterogeneity that existed in the three forest plots pooling the three outcome measures (level of functioning, quality of life, and symptoms) should be considered in analysing the results of this review. This heterogeneity might be attributed to variations in sample size, the way outcomes were measured, variation in the studies’ design, and the follow-ups periods of included studies. It is a challenge to draw firm final conclusions from the results of this meta-analysis for several reasons. First, the methodological shortcomings of the included studies, such as small sample sizes, unclear description of the study design, incomplete reporting of randomization procedures, and the lack of clear definition and description of the strengths-based approach. Second, relatively few primary studies were conducted to test the efficacy of the strengths-based approach. Third, the use of the term ‘severe mental illness’ in the primary studies is too vague; this population is diverse and an investigation of subgroups of this population would yield more accurate and specific findings. Finally, the high heterogeneity among the included studies limits the conclusions one can draw from this review.

Excluding outlier studies that contributed to the substantial heterogeneity in this review resulted in the following observations. In the functional capacity plot, Chamberlain [19] was the outlier study and removing it from the forest plot resulted in reduction of heterogeneity. However, no significant effects were reported between the strengths-based approach and other service delivery models after removing this outlier study as shown in Figure 3. Barry et al. [18] was considered the outlier study in the forest plot of symptoms (related to heterogeneity in sample size and of very negative results). Excluding it from the forest plot resulted in reduction in the heterogeneity and changed the overall effect from favouring the control before exclusion to no significant difference after the exclusion (from p < 0.0001 to p = 0.93) as shown in Figure 6.

### Overall quality of the evidence

The included primary studies are of low methodological quality; the RCTs lack any information about randomisation, allocation concealment and blinding. Moreover, the authors of primary studies failed to provide a clear and definite description of the strengths-based approach making it difficult to distinguish it from other delivery models.

### Strengths of this review

This is the first systematic review with meta-analysis evaluating the impact of the strengths-based approach as a service delivery model of functional capacity, quality of life, and symptoms.

### Limitations of this review

Restricting the language of included primary studies to English due to feasibility and resources issues is considered a limitation in this review (and a potential bias as well). In addition, the substantial heterogeneity and low quality of included studies added to the weakness of findings, particularly with relation to implications for practice.

### Implications for research

It is necessary for future studies in this area to consider and present their methods more clearly, particularly the description of the strengths-based approach. Moreover, presentation of all numerical data to guide prospective reviews is recommended.

## Conclusion

Based upon evidence of moderate quality, this review suggests there is no effect of the strengths-based model of service delivery in level of functioning and quality of life in adults diagnosed with severe mental illness. The number of trials is low. Therefore, further evidence is required to ascertain the impact of the strengths-based approach in community mental health.

## Additional files

Additional file 1:
**MEDLINE (Ovid) serach strategy which was adapted for other data base seraching.**
Additional file 2: Table S1. Characteristics of included studies and risk of bias.

## Abbreviations

RCT: Randomized Controlled Trial
MeSH: Medical Subjects’ headings
DSM: The Diagnostic and Statistical Manual of Mental disorders
ICD: International Classification of disease
RevMan: Review Manager
PRISMA: Preferred Reporting Items of Systematic reviews and meta-analyses
JBI: Joanna Briggs Institute
EPOC: Effective Practice and Organisation of Crae
IADLL: Instrumental Activity of daily Living
GAF: Global assessment of functioning
OQOLG: Oregon Quality of Life Questionnaire
BPRS: the Brief Psychiatric Rating Scale
UCDI: Uniform Client Data Inventory
CI: Confidence Interval
SPSS: Software Package of Statistical Analysis
SE: the Standard Error
SMD: the Standardised Mean Difference
MD: Mean difference
SD: the standard Deviation
(df): Degree of freedom
