# Supplementary material for: The strengths based approach as a service delivery model for severe mental illness: a meta-analysis of clinical trials
Source: BMC Psychiatry. 2014 Aug 29;14:243. doi: 10.1186/s12888-014-0243-6 (PMC4154523; doi:10.1186/s12888-014-0243-6)
Supplement: Additional file 1: — MEDLINE (Ovid) serach strategy which was adapted for other data base seraching. [file 12888_2014_243_MOESM1_ESM.doc]

MEDLINE (Ovid) serach strategy which was adapted for other data base seraching.

1. Strengths model. mp.

2. Strengths perspective.mp.

3. Case Management/ or strengths case management.mp.

4. Assets based approach.mp.

5. Developmental acquisition model.mp.

6. Strengths based approach.mp.

7. 1 OR 2 OR 3 OR 4 OR 5 OR 6

8. Psychotic Disorders/

9. Mental Disorders/

10. Persistent mental illness.mp.

11. chronic psychosis.mp.

12. 8 OR 9 OR 10 OR 11

13. 7 and 12

14. Randomised controlled trials.mp.

15. Intervention Studies/

16. experiment.mp.

17. Controlled Clinical Trial/

18. 14 and 15 and 16 and 17.

19. 7 and 12 and 18
