# Supplementary material for: The strengths based approach as a service delivery model for severe mental illness: a meta-analysis of clinical trials
Source: BMC Psychiatry. 2014 Aug 29;14:243. doi: 10.1186/s12888-014-0243-6 (PMC4154523; doi:10.1186/s12888-014-0243-6)
Supplement: Additional file 2: Table S1. — Characteristics of included studies and risk of bias. [file 12888_2014_243_MOESM2_ESM.doc]

**Table (S1) characteristics of included studies and risk of bias**

### Barry et al. (2003), quasi-experimental study

| **Methods** | This study was a subset of a larger longitudinal study. A quasi-experimental design was used where the strengths-model was compared to assertive community treatment (ACT) for patients with serious mental illness. Assessments were conducted at baseloine and every 6 months thereafter 2 years. |
| --- | --- |
| **Participants** | Individuals with serious and persistent mental illness were included if they were diagnosed with a psychotic disorder according to the DSM III-R, admitted for 150 or more days of hospitalization in the past year or 5 or more admissions during the past year . The average age of participants was 49.5%, both sexes were included in this study, ethnicity was predominantly white, 81 individuals were in the strengths model group, 93 in the ACT control group. |
| **Interventions** | The strengths-model was described in this study was compared with the assertive community treatment in the strengths-model, patient is assigned to one case manager, while in the assertive community treatment, the case manager is in charge of more than one patient. Moreover, the strengths-model was described as; the focus of the model is on individual strengths rather than deficits; engagement between the case manager and the service user is of primary importance; client is responsible for setting his goals and patient-self-determination is central to this model; contacting clients takes place in the community; and the ability to grow is inherited in people with mental illness. |
| **Outcomes** | Positive and negative symptoms, global life satisfaction, activity of daily living, total inpatient days, total outpatient visits, inpatient medical, psychiatric, and nursing home days. |

**Bjorkman et al. (2002)**

| **Methods** | A randomised controlled trial where randomisation was conducted using random procedure in SPSS program. Case finding procedure was used to allocate eligible participants from the psychiatric services, primary health care services, social services, and the regional social insurance offices. The strengths-based case management was compared to the standard care. |
| --- | --- |
| **Participants** | Inclusion criteria to recruit participants in study were; diagnosis of serious mental illness causing impairment and difficulties in functioning in social relationships, housing, or work situation for more than 2 years. Participants’ age ranged from between 18 and 55 years, individuals with substance misuse disorder were excluded. Participants were allocated to the study group where the strength-case management was conducted (n=33) and a control group (standard care group, n=44). |
| **Interventions** | The strengths-case management services in this study were implemented by two registered nurses and two social workers. The average case load was nine clients and an average of 1.45 direct client contacts was conducted per week. Service was supervised by a psychiatrist and a psychologist. The strengths-based service placed moderate emphasis on skills training, low emphasis on integration of services, and high emphasis on clients input on the service where the client shares planning as well as implementing the service. |
| **Outcomes** | Symptoms, psychosocial functioning, social network, quality of life, and client satisfaction. |

| **Bias in Bjorkman at al. (2002)** | **Authors' judgement** | **Support for judgement** |
| --- | --- | --- |
| Random sequence generation (selection bias) |  | The random selection of the sample was performed by one of the study’s researchers using a computer file comprising the original cohort. |
| Allocation concealment (selection bias) |  | The random allocation procedure was made by the random number procedure in the SPSS program |
| Blinding of participants and personnel (performance bias) |  | No information was provided |
| Blinding of outcome assessment (detection bias) |  | The interviewers were formally blinded to the group allocation of the clients |
| Incomplete outcome data (attrition bias) |  | Of the 77 clients entering the study, data were available for 66 clients at the 18 months follow up and 64 clients at the 36 months follow up. |
| Selective reporting (reporting bias) |  | All predefined outcomes were reported where no data was missing. |

**Chamberlain (1991)**

| **Methods** | Random assignment to the experimental and control group was performed by using a table of random numbers. The strengths model was compared with traditional community services (chemotherapy and psychotherapy services). |
| --- | --- |
| **Participants** | All subjects were patients from the adult psychiatric unit and are, therefore, 18 years of age or older. Proportion of male to female was nearly equal. The race and ethnic composition of the sample; white, black and Hispanic. 25 subjects were in the experimental group and 24 in the control group. |
| **Interventions** | The intervention focused on building individual strengths through community teaching of problem solving using modelling, role playing, goals’ setting, and acquisition of community resources. The functional elements of the model are; both the client and the case manager assess client’s non-clinical strengths; mutual contracts between the client and the case manager are established where goals are prioritised; the role of the case manager is to identify resources and helping the client to acquire these resources; of social problem solving can be acquired through variety of techniques which are inherent in this model; case manager monitors client’s achievement of the goals as well as negotiating with him to help achieving these goals. |
| **Outcomes** | The number of hospital readmissions, quality of life, level of functioning, resource utilization, and goal set and achieved by life domain |

| **Bias in Chamberlain (1991)** | **Authors' judgement** | **Support for judgement** |
| --- | --- | --- |
| Random sequence generation (selection bias) |  | A table of random numbers was used to randomly assign participants to experimental or control groups |
| Allocation concealment (selection bias) |  | No data was provided |
| Blinding of participants and personnel (performance bias) |  | No available data |
| Blinding of outcome assessment (detection bias) |  | Not clear |
| Incomplete outcome data (attrition bias) |  | No data was reported about attrition |
| Selective reporting (reporting bias) |  | All predefined outcomes were reported. |

**Macias et al. 1994**

| **Methods** | A randomise controlled trial where 41 participants were randomised by the mental health centre to experimental case management group and a control group. Measures were taken at baseline then 18 months after implementation of the program. |
| --- | --- |
| **Participants** | 41 seriously mentally ill adults were eligible for the case management services ( who have serious and persistent mental illness, patients with primary diagnosis of mental retardation or substance abuse were excluded from this study. 32.5% of the sample was less than 28 years of age, 32.5% were between the age of 28 and 45, and 35% were over 45 years. Both sexes were included. The study sample was entirely Caucasian. |
| **Interventions** | The strengths-based case management emphasized clients’ goal setting as well as strong client-practitioner relationship. The case managers were trained to provide linkage as well as brokerage of social and medical services. The case managers provided full time services and were supervised by a director. Case management team shared a case load of 20 clients as the strengths-based case management to ensure close contact between the service users and case managers |
| **Outcomes** | Depression, anxiety, and somatic complains. global mental and physical health, problems with thinking, emotional problems, level of social support, competence in daily living, and service satisfaction. Daily life functioning. level of dependency, rationality, money management, family relations, friendship relations, education, vocational/daily activity, physical health, substance use, and legal problems |

| **Bias in Macias et al. (1994)** | **Authors' judgement** | **Support for judgement** |
| --- | --- | --- |
| Random sequence generation (selection bias) |  | Not described |
| Allocation concealment (selection bias) |  | No data was provided |
| Blinding of participants and personnel (performance bias) |  | No information was provided |
| Blinding of outcome assessment (detection bias) |  | No available data |
| Incomplete outcome data (attrition bias) |  | One participant refused participation, resulting in a final sample of 41 consumers. 18 out of 20 for the case management and 16 out of the 21 for the control group had been retained at the time of the 1991 interviewa.5 of the 7 consumers lost to attrition were replaced by new consumers who entered the community support program |
| Selective reporting (reporting bias) |  | Predefined outcomes were reported |

**Modrcin et al. (1988)**

| **Methods** | A randomised controlled trial where the developmental acquisition model (strengths model) was compared to traditional case management services. |
| --- | --- |
| **Participants** | A total of 89 individuals were referred for service during this time and were randomly assigned to the experimental group to receive case management service (the developmental acquisition model or the control group which received the normal intervention case management services. Of the 89 people randomly assigned to the 2 case management approaches, 38 didn't participate. The 38 were evenly divided (19 each) between the 2 groups. |
| **Interventions** | As described in the study, the procedure of implementing the intervention was guided by 1-250 page manual (Modrcin, Rapp, and Chamberlain, 1985) and upon request the author stated that this model was withdrawn and replaced by the strengths model- a recovery oriented approach to mental health services by Rapp and Goscha (2012). However, the study presented a synopsis from this manual “the clients often have limited information about community services and resources; the case manager provides it. Client’s characteristically experience profound anxiety when confronting new tasks and challenges; the case manager supports and encourages. Clients frequently lack information or skills in basic aspects of independent living skills to form and sustain social relationships; the case manager offers a relationship, models and teaches social skills, and helps the client generalise that to other persons. Clients sometimes experience painful crises that place their; live in turmoil and increase symptomatic behaviour; the case managers are a first line of intervention to minimise the potential for regression or relapse”. |
| **Outcomes** | Quality of life, medication compliance, hospitalization, employment, and vocational training |

| **Bias in Modrcin et al. (1988)** | **Authors' judgement** | **Support for judgement** |
| --- | --- | --- |
| Random sequence generation (selection bias) |  | The authors just reported that participants were randomly assigned to the arms of the study, with no data about the random sequence generation. |
| Allocation concealment (selection bias) |  | No data was provided |
| Blinding of participants and personnel (performance bias) |  | Not stated in the paper |
| Blinding of outcome assessment (detection bias) |  | Not described in the paper |
| Incomplete outcome data (attrition bias) |  | No data was provided about attrition |
| Selective reporting (reporting bias) |  |  |
